# Microsurgical treatment of the radicular arteriovenous fistula with multiple feeders at the craniocervical junction

**DOI:** 10.3171/2025.7.FOCVID2526

**Published:** 2025-10-01

**Authors:** Satoshi Matsuo, Kenta Hara, Akifumi Yokomizo, Toru Hasegawa, Hidenori Yoshida, Kiyotaka Fujii

**Affiliations:** Department of Neurosurgery, Fukuoka Tokushukai Hospital, Fukuoka, Japan

**Keywords:** craniocervical junction, radicular arteriovenous fistula, subarachnoid hemorrhage

## Abstract

Arteriovenous fistula of the craniocervical junction is a rare vascular malformation. Although a precise understanding of its angioarchitecture is essential for appropriate and curative treatment, a preoperative diagnosis can be difficult owing to its complexity. Intraoperative findings may provide more clues to the definitive diagnosis. In this video, the authors present an operative technique for exposing a radicular arteriovenous fistula at the craniocervical junction fed by the radicular and spinal pial arteries draining into the common intradural vein. This fistula was definitively diagnosed based on intraoperative findings and treated by disconnecting the common draining vein.

The video can be found here: https://stream.cadmore.media/r10.3171/2025.7.FOCVID2526

## Transcript

The following represents a case of a radicular arteriovenous fistula (AVF) at the craniocervical junction (CCJ) fed by the radicular and spinal arteries draining into a common intradural vein. CCJ AVFs often possess complicated angioarchitecture; therefore, detailed preoperative and intraoperative evaluations are essential for planning treatment strategies.

### 0:44 Case Presentation.

A 57-year-old man was admitted to our emergency department with a sudden headache and posterior neck pain. His Glasgow Coma Scale score was 15, and he was neurologically intact.

### 0:56 Neuroimaging Findings.

CT images revealed a subarachnoid hemorrhage (SAH), predominantly in the posterior cranial fossa. CT angiography showed no intracranial aneurysm but a vascular malformation suggestive of arteriovenous (AV) shunt disease at the CCJ. Right vertebral angiography (VAG) showed an AV shunt fed by the right C2 radicular artery, which drained the region through the anterior spinal vein intradurally and into the vertebral venous plexus epidurally. This slide shows the drainage route of the fistula. Left VAG revealed that the AV shunt, as described earlier, was fed by the pial arteries from the anterior spinal artery (ASA) and drained into the anterior spinal vein through the same coronary vein, as mentioned earlier. Fusion images with CT angiography and MR cisternography revealed that the AV shunt was located in the intradural space, close to the right C2 ventral root.

### 1:59 Preoperative Diagnosis.

This schematic drawing showed the angioarchitecture of the AVF based on the preoperative examinations. Because the preoperative images could not identify the exact location of the shunt point, preoperative diagnoses of radicular AVF, concurrent dural and perimedullary AVF, and dural AVF with a pial feeder were made.

### 2:24 Alternatives and Decision-Making.

To prevent recurrent SAH, we opted to treat the AVF.

Endovascular^1,2^ and microsurgical treatments^3,4^ and their combination^5^ are used to treat CCJ AVFs. Considering that the branches from the ASA fed to the AVF, endovascular embolization carried a high risk of ischemic complications. Therefore, we planned microsurgical treatment.

### 2:49 Key Surgical Steps.

These are key steps of this surgery. The patient was placed in the prone position in the hybrid operating room. Under general anesthesia, the left brachial artery was cannulated to perform intraoperative left VAG to examine the feeder from the ASA. A skin incision and craniotomy were performed as shown in these slides.

### 3:10 Surgical Procedure.

Here is an overview of surgical exposure. The top of the head was placed at the bottom of the screen. The SAH was washed out almost completely. After the dentate ligament was identified just rostral to the C2 nerve root, the medial part of the ligament was incised. A small vessel was identified along the C2 ventral root, probably a feeder from the ASA. It coursed caudally along the C2 ventral root and joined the dilated radicular vein, which appeared to be a single common draining vein. At the dural penetration of the C2 ventral root, other small vessels, probably feeders from the C2 radicular artery, were identified. To enlarge the surgical field, the remaining dentate ligament was coagulated, stitched with 9-0 suture, and incised. The distal end of the ligament was then retracted. A shunt point was observed at the C2 ventral root. A single common draining vein was confirmed. ICG videoangiography was performed, showing that the C2 ventral root was entangled with small vessels. An intraoperative left VAG was performed, demonstrating that the AVF was fed by pial arteries branching from the ASA. A common single drainer was firmly attached to the C2 ventral root so that a clip was applied to the vein with the C2 nerve root distal to the fistula. The proximal portion was slightly dilated. Although it did not look like a varix, another clip was applied to the C2 radicular artery with C2 ventral root immediately after they penetrated the dura. We confirmed that both clips were sufficiently deep to trap the AVF and draining vein. Intraoperative left VAG showed that the AVF had disappeared, while the ASA was preserved. The vessels between clips were coagulated and incised. ICG videoangiography also verified the disappearance of the AVF. Based on these findings, we concluded that the right C2 radicular artery and pial feeders branching from the ASA drained into the common radicular vein through a shunt located on the C2 ventral root. These schematic drawings show the position of the clips. This patient was finally diagnosed with a radicular AVF. Complete hemostasis was achieved, and the wound was closed as usual.

### 5:59 Postoperative Course.

Postoperative CT and MRI revealed no hemorrhage or infarction. CT angiography showed the disappearance of the AVF. Digital subtraction angiography also showed the disappearance of the AVF while preserving the ASA. The patient’s postoperative course was uneventful. He remained neurologically intact. After discharge, the patient’s condition returned to normal. At the latest follow-up, 2 years postoperatively, MRI confirmed no recurrence of the fistula. Thank you.

## Acknowledgments

We thank Editage for English-language editing.

## Disclosures

The authors report no conflict of interest concerning the materials or methods used in this study or the findings specified in this publication.

## Author Contributions

Primary surgeon: Matsuo. Assistant surgeon: Hara, Yokomizo, Hasegawa. Editing and drafting the video and abstract: Matsuo, Hara, Yokomizo, Yoshida, Fujii. Critically revising the work: Matsuo. Reviewed submitted version of the work: Matsuo. Approved the final version of the work on behalf of all authors: Matsuo. Supervision: Hasegawa, Yoshida, Fujii.

## Supplemental Information

### Previous Presentations

Portions of this work were presented as a poster presentation at the 34th Annual Meeting of the North American Skull Base Society, New Orleans, LA, February 13–16, 2025.

### Patient Informed Consent

The necessary patient informed consent was obtained in this study.
